# A Systematic Review of Cognitive Behavioral Therapy and Behavioral Activation Apps for Depression

**DOI:** 10.1371/journal.pone.0154248

**Published:** 2016-05-02

**Authors:** Anna Huguet, Sanjay Rao, Patrick J. McGrath, Lori Wozney, Mike Wheaton, Jill Conrod, Sharlene Rozario

**Affiliations:** 1 Center for Research in Family Health, IWK Health Centre, Halifax, Nova Scotia, Canada; 2 Department of Community Health & Epidemiology, Dalhousie University, Halifax, Nova Scotia, Canada; 3 Annapolis Valley Health, Kentville, Nova Scotia, Canada; 4 Department of Psychiatry, Dalhousie University, Halifax, Nova Scotia, Canada; 5 Departments of Pediatrics and Science, Dalhousie University, Halifax, Nova Scotia, Canada; 6 Nova Scotia Health Authority, Halifax, Nova Scotia, Canada; University of South Australia, AUSTRALIA

## Abstract

Depression is a common mental health condition for which many mobile apps aim to provide support. This review aims to identify self-help apps available exclusively for people with depression and evaluate those that offer cognitive behavioural therapy (CBT) or behavioural activation (BA). One hundred and seventeen apps have been identified after searching both the scientific literature and the commercial market. 10.26% (n = 12) of these apps identified through our search offer support that seems to be consistent with evidence-based principles of CBT or BA. Taking into account the non existence of effectiveness/efficacy studies, and the low level of adherence to the core ingredients of the CBT/BA models, the utility of these CBT/BA apps are questionable. The usability of reviewed apps is highly variable and they rarely are accompanied by explicit privacy or safety policies. Despite the growing public demand, there is a concerning lack of appropiate CBT or BA apps, especially from a clinical and legal point of view. The application of superior scientific, technological, and legal knowledge is needed to improve the development, testing, and accessibility of apps for people with depression.

## Introduction

Depression is one of the most common mental health disorders [1] which often begins in adolescence and if left untreated, may persist into adulthood [2]. It ranks 4th in the global burden of disease [3]and is of significant economic cost to society[4]. Cognitive Behavioural Therapy (CBT) and Behavioural Activation (BA) are now an accepted evidence-based first-line treatment for depression [5]. Both CBT and BA have meta-analytic level of evidence in the treatment of depression[6,7]. Periodic face-to-face sessions between therapist and patient have been the most traditional medium to deliver CBT and BA. However, with population estimates of Major Depression at 6.7% and even higher for Non-Major Depression [1], it is unlikely that this traditional approach can reach everyone.

More recent research indicates that depression can be treated successfully with CBT and BA based self-help interventions delivered over the Internet [8,9,10]. This type of therapy is suited for digital delivery as demonstrated by the fact that there are more Internet-based studies on CBT/BA than on other evidence-based models (e.g., Interpersonal Therapy or Acceptance and Commitment Therpy). There is a strong case in healthcare for addressing access to CBT or BA through the use of technology, with mobile applications (apps) being one possible means of delivery. Apps could be especially useful in early treatment of depression in young people who report high levels of smartphone device use [11].

Smartphone use is a growing phenomenon [12] and has the advantage of being accessible, mobile, and easy to operate, with decreasing cost of use. Smartphones have been used to facilitate the delivery of healthcare interventions including treatment of mental health conditions [13]. The number of apps intended to help people cope with depression is increasing rapidly, especially in the commercial marketplace [14,15]; however the development process, usability, feasibility, and efficacy of these apps developed in the commercial marketplace are rarely assessed or reported. The quality of the available apps has not been the subject of any systematic reviews, until now.

It is vital to perform a systematic review of apps for depression to identify what currently available apps are based on strong and recommended evidence models for depression. Evaluating the available apps can inform future development of effective smartphone delivered intervention for depression. The purpose of this systematic review was twofold: (1) To identify all currently-available native apps that provide information, support or treatment for depression; (2) To evaluate CBT or BA self-help (either guided or unguided) apps on their usefulness, usability, and integration and infrastructure, as recommended by Chan et al. [16]. Usefulness was determined by evaluating how accurately each CBT/BA app tapped into the core of the CBT and BA models, and by exploring whether the efficacy or effectiveness of the CBT/BA apps have been proven or not. Usability was evaluated by comparing each CBT/BA app to a list of heuristics, and integration and infrastructure was evaluated by looking whether the CBT/BA apps included a privacy policy and addressed safety issues.

The results of this review can assist care providers in choosing appropriate apps for the treatment or research of depression. The review will also identify areas for future development to effectively provide CBT or BA for depression through smartphones.

## Methods

### Inclusion and Exclusion criteria

We included in our review those apps that met the following inclusion criteria: (1) the app description stated that they provide treatment or support for depression as its exclusive goal; (2) the app was publically available for download within Canada at the time this review was performed (December, 2015), and consequently also fully available for evaluation by the research team; (3) the app was defined as a native app (i.e., developed for one particular mobile device and installed directly onto the device itself) compatible with smartphones. We excluded from the review those apps which specifically addressed depressed subpopulations (e.g., depressed people with diabetes, postpartum depression) because they have special health care needs that require different care. We also excluded those apps that were designed to support health care professionals working with depressed populations because these apps are addressed to a different audience. We excluded web-based/Internet-enabled apps only accessible via the mobile device’s Web browser because they are very challenging to identify in a systematic way. Finally, we also excluded those apps which were only available in a non-English language.

### Search strategy

The apps included in this review were identified by searching both the scientific literature and commercial marketplace.

#### The search of the scientific literature

**Fig 1:**
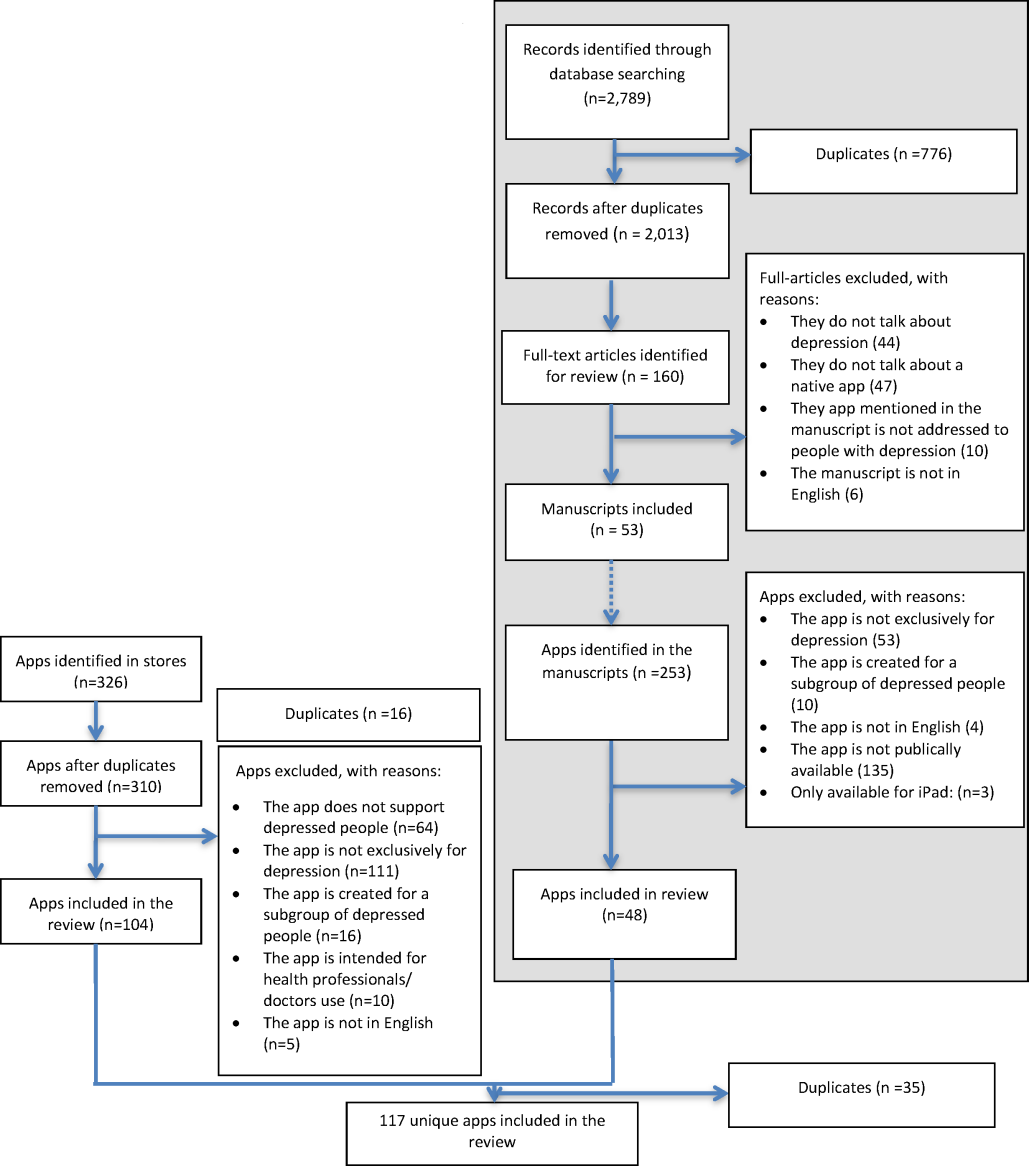
Flowchart of the screening process.

The following databases from health sciences and computer science were searched: IEEE, ACM Digital Library, EMBASE, PubMed (Medline), PsychINFO, and Web of Science. A library information specialist created the database-specific search strategies by combining population-specific term (i.e., depression) and terms related to technical delivery (i.e., app, smartphone, mobile phone, cell phone, text message, iphone, and android), narrowing the results to those studies related to depression and mobile apps. Search strategy in S1 Appendix displays the strategy for retrieving relevant manuscripts from PubMed. The library information specialist did the search in November 2015. During the first level of screening, two reviwers (AH, SR) independently assessed a random selection of 15% of the titles and abstracts retrieved from search (350 electronic search results) to determine inter-rater agreement on inclusion and exclusion criteria. With substantial levels of agreement (kappa = 0.69) observed [17], the remaining titles and abstracts were screened by only one reviewer (SR). At the second level of screening, potential relevant full- text articles were reviewed and a random selection of 30% of articles (a subset of 50 articles) were independently assessed by two reviewers (AH, SR). Articles were excluded at this stage from further consideration for a number of reasons (i.e., article did not talk about depression, article did not make mention of any native app, the app mentioned in the article was not addressed to people with depression, the manuscript was not written in English). With substantial levels of agreement observed at this second level of screening (kappa = 0.85) [17], the remaining full-text articles were reviewed by only one reviewer (SR). The 53 manuscripts included at this stage mentioned a total of 253 native apps for people with depression. Two independent reviewers (SR, AH) independently evaluated whether a random selection of 50% of these 253 apps (n = 125) meet the eligibility criteria based on our inclusion/exclusion criteria. With almost perfect agreement observed at this third level of screening (kappa = 0.92) [17], the remaining apps were reviewed by only one reviewer (SR). Contact was made with corresponding authors to request access to any apps described in a manuscript where there was no information provided on public access for downloading. Discrepancies at any level of screening were resolved by consensus among reviewers. See Fig 1 for details about the screening process.

#### The search of the commercial market place

The search was restricted to apps available through the two most popular mobile phone platforms, The Canadian Apple App Store and Android Market (Google Play). The search was made in November 2015 using ‘depression’ as the search query. One reviewer (JC) searched the stores to identify all of the available apps, and two reviewers (AH, JC) independently evaluated each identified unique app for eligibility based on our inclusion/exclusion criteria. The level of agreement between both independent reviewers using the Cohen’s Kappa was 0.89. Discrepancies were resolved through discussion. See Fig 1 for further details.

### Data extraction

The apps retrieved by our searches were categorized by two independent reviewers (AH, JC) according to the type(s) of support that they offered to the users. The categories, defined a priori, included: self- tracking tools, education, social support, CBT/BA treatment, state induction, diagnostic/screening tools, and miscellaneous. One app could be categorized into different types of self-help apps when the app included more than one type of support. All the apps included in the review were available in the app stores, regardless of where they were identified (i.e., scientific literature vs commercial market). The app description displayed in the stores and any available description provided in the manuscript was the only information used by the reviewers to base their decisions on which category each app fell into. The level of agreement between the two reviewers when categorizing the apps, using the Cohen’s kappa, was 0.92, indicating almost perfect agreement [17]. When reviewers were in disagreement, they discussed it, and came to an agreement. When an agreement could not be reached, a third reviewer was called upon (SR). For those apps that were classified as CBT/BA the following information was extracted: accessibility (i.e., iTunes, Google Play, scientific literature), cost, and indicators of popularity (i.e., for the apps identified through the Google Play store, the number of times an app has been downloaded to an android phone; for the apps identified through the Google Play store or the iTunes store, the number of users that have rated the app on a scale of 1 to 5 as well as the average satisfaction rate provided by users; although both types of information are only available when there is a large, unspecified amount of users that have rated the app).

#### Assessment of CBT/BA apps

Since our primary focus of attention was CBT or BA only those apps that offered this type of treatment were downloaded for full evaluation. When both a paid and free version of an app was available, the version requiring payment was purchased and used, while the free version was excluded. This was done to ensure that the most comprehensive version of the app was considered. In accordance with Chan et al [16], who have recently proposed a framework to evaluate mobile mental health apps, we evaluated each app on three dimensions using the following criteria:

*Usefulness*: To determine the usefulness of the apps, the validity and accuracy (does the app actually offer CBT or BA?), and effectiveness (is the app clinically effective–with demonstrated improved outcomes- for people with depression?) criteria were used. To evaluate whether the app actually offers CBT or BA, an experienced academic CBT clinician (SR) evaluated the apps for their level of fidelity to theoretical CBT and BA principles by exploring what extent the apps included the core ingredients of these models. The evaluator has extensive experience in training CBT therapists and devising CBT clinical programmes. The core ingredients for CBT and BA were derived by consulting with two academic experts and one CBT clinician, as well as reviewing the literature for CBT and BA models in the treatment of depression [18,19]. The following were considered as the core ingredients of a CBT approach for depression: 1) education about depression; 2) explanation of the model, 3) depression rating, 4) monitoring cognitions, 5) monitoring emotions, 6) monitoring physical sensations, 7) monitoring behaviours, 8) conceptualization, 9) behavioural techniques, and 10) cognitive techniques. The following were considered as the core ingredients of the various BA approaches: 1) education about depression, 2) explanation of the model, 3) depression rating, 4) activity monitoring, 5) giving each activity a rating for pleasure, 6) giving each activity a rating for mastery, 7) activity scheduling of pleasant behaviours, and 8) activity scheduling of avoided behaviours. The expert evaluated each app against each core ingredient on a 0–2 scale where 0 meant that the core ingredient was not integrated at all into the app, and 2 meant that the core ingredient was completely integrated. Table 1 displays the scoring system devised for rating of the apps against each core ingredient. For each app, a percent total score (sum of item scores/maximum possible score * 100), representing the level of adherence of the app to the theoretical principles of CBT and BA approaches, was then calculated. To evaluate the effectiveness of the apps, we cross-referenced with apps identified in the scientific literature to see whether there was any efficacy or effectiveness study on apps included in the review.

**Table 1:** System to grade the level of adherence to the theoretical principles of CBT and BA.

		Item		
	Core features	0	1	2
**Behavioural activation**				
	Education about depression	None	Some	Clear explanation
	Explanation of the model	None	Some	Clear explanation
	Depression rating	None	Some	Formally rated (e.g., on a 0–10 scale)
	Activity monitoring	None	Some	Formally self-monitoring (e.g., through a diary)
	Activity monitoring: Pleasure rating	None	Some	Formally rated (e.g., on a 0–10 scale)
	Activity monitoring: Mastery rating	None	Some	Formally rated (e.g., on a 0–10 scale)
	Activity scheduling of pleasant behaviours	None	Some	Formally rated (e.g., on a 0–10 scale)
	Activity scheduling of avoidancebehaviours	None	Some	Formally rated (e.g., on a 0–10 scale)
**Cognitive-behavioural treatment**				
	Education about depression	None	Some	Clear explanation
	Explanation of the model	None	Some	Clear explanation
	Depression rating	Some	Some	Formally rated (e.g., on a 0–10 scale)
	Monitoring cognitions	None	Some	Thoughts and beliefs monitored
	Monitoring emotions	None	Some	Specific emotions monitored
	Monitoring physical sensations	None	Some	Specific physical sensations monitored
	Monitoring behaviours	None	Some	Specific behaviours monitored
	Conceptualization	None	Someelements	Adequate problem formulation
	Cognitive techniques	None	Some	Systematic use of technique
	Behavioural techniques	None	Some	Systematic use of technique

*Usability*: The usability of the app (can the user easily–or with minimal training- use and understand the app?) was used to evaluate this dimension. Most apps retrieved from our searches have been developed by small businesses or sole proprietors outside of academic settings, and little information is available on the app development process or evidence of formal usability testing. For this reason, a user experience designer (MW), who regulary performs expert reviews on mobile apps and websites, where he applies heuristics and professional experience to evaluate user interfaces and suggest design improvements, evaluated the usability of the apps. He evaluated the user interface of each app using a common list of usability heuristics proposed by Nielsen & Mack [20]. The usability expert rated each app on a scale of 1 to 5 (1 = poor, 5 = excellent) against each usability heuristic (see Table 2 for the set of heuristics). A percentage total score (sum of item scores/maximum possible total score * 100) was then calculated, indicating the extent to which the user interface of the app met the usability heuristics.

**Table 2:** Heuristics used to assess usability of the apps.

Heuristic	Description
Visibility of system status	The system should always keep users informed about what is going on, through appropriate feedback within reasonable time.
Match between system and the real world	The system should speak the users' language, with words, phrases and concepts familiar to the user, rather than system-oriented terms. Follow real-world conventions, making information appear in a natural and logical order.
User control and freedom	Users often choose system functions by mistake and will need a clearly marked "emergency exit" to leave the unwanted state without having to go through an extended dialogue. Support undo and redo.
Consistency and standards	Users should not have to wonder whether different words, situations, or actions mean the same thing. Follow platform conventions.
Error prevention	Even better than good error messages is a careful design which prevents a problem from occurring in the first place. Either eliminate error-prone conditions or check for them and present users with a confirmation option before they commit to the action.
Recognition rather than recall	Minimize the user's memory load by making objects, actions, and options visible. The user should not have to remember information from one part of the dialogue to another. Instructions for use of the system should be visible or easily retrievable whenever appropriate.
Flexibility and efficiency of use	Accelerators—unseen by the novice user—may often speed up the interaction for the expert user such that the system can cater to both inexperienced and experienced users. Allow users to tailor frequent actions.
Aesthetic and minimalist design	Dialogues should not contain information which is irrelevant or rarely needed. Every extra unit of information in a dialogue competes with the relevant units of information and diminishes their relative visibility.
Help users recognize, diagnose, and recover from errors	Error messages should be expressed in plain language (no codes), precisely indicate the problem, and constructively suggest a solution.
Help and documentation	Even though it is better if the system can be used without documentation, it may be necessary to provide help and documentation. Any such information should be easy to search, focused on the user's task, list concrete steps to be carried out, and not be too large.

*Integration and infrastructure*: Privacy and safety were the criteria used to evaluate this dimension. To evaluate privacy, an evaluator (SR) looked into whether the apps provided users with a privacy policy (within the apps themselves or on a website linked to the app). If a privacy policy was available the evaluator assessesed the scope and the level of transparency of the policy as done by Sunyaev et al. [21]. To this end, the evaluator determined whether the policy addressed the following content categories important to users: type of information collected (e.g., operational, behavioral, sensitive), rationale for collection (i.e., app operation, personalization, secondary use), sharing of information (i.e., service provision, social interaction, third party), and users controls (i.e., supervision, notification, correction). To evaluate safety, an evaluator (SR) explored whether the apps had any mechanisms in place to handle high risk of suicidality (e.g. providing emergency contact information whenever the app detects a user is at high risk for committing suicide).

## Analysis Plan

Basic summary statistics including counts and percentages were used to describe the characteristics of the apps. Spearman’s correlation coefficient was used to explore whether a relationship may exist between the adherence of the user interface to Nilsen’s principles of usability and adherence to the core principles underlying CBT and BA. Spearman’s correlation coefficients were also used to explore whether adherence to the core principlies underlying CBT and BA and adherence to Nilsen’s principles of usability is related with any indicator of popularity and acceptability (i.e., average rating of satisfaction, number of reviews and number of downloads).

## Results

### Search

Our search of commercial marketplace identified a total of 310 unique apps. One hundred and four of these apps identified in the commercial marketplace meet our inclusion/exclusion criteria. The literature search yielded 2,789 abstracts, and 160 full text manuscripts were reviewed at the full-text level. Fifty-three out of 160 were relevant for our review because all them mention at least one native app addressed to people for depression. Many of these manuscripts identified as relevant for our review were reports or reviews reporting on multiple apps. For example, Shen et al. [14], has recently conducted a systematic review to identify and characterize all the apps available in the app stores to support people with depression, their families and health care professionals, based on the store description. The 53 manuscripts [9,14,15,22,23,24,25,26,27,28,29,30,31,32,33,34,35,36,37,38,39,40,41,42,43,44,45,46,47,48, 49,50,51,52,53,54,55,56,57,58,59,60,61,62,63,64,65, 66,67,68,69,70,71] identified as relevant to the review made mention of a total of 48 unique apps that met our inclusion/exclusion criteria. Thirty-five of these 48 apps were also identified through our search of the commercial marketplace. See Fig 1 for a flowchart of the screening process of the apps.

### App characteristics

Out of the total 117 apps, 36 apps (30.77%) were available on iOS only, 74 (63.25%) were available on Android only, and 7 (5.98%) were available across both platforms. The most typical type of self-help support delivered through these 117 apps was education (n = 32, 27.35%) and diagnostic/screening support (n = 30, 25.64%), followed by state induction (n = 18, 15.38%). The least typical types of self-help support delivered through these 117 apps were tracking (n = 10, 8.55%) and social support (n = 3, 2.56%). Twelve of these 117 apps (10.26%) were classified by the reviewers as delivering CBT or BA; these CBT/BA apps were identified in the description by their developers as CBT or BA apps or they seemed to offer CBT or BA based on their general description (Table 3).

**Table 3:** All apps for depression included in the review.

Type of Self-Help App	Screening	Number of Apps^a^	Total Number of apps (%)^b^	Name of the Apps
**Tracking**	Commercial Marketplace	6	10 (8.55)	Depression Test, Depression Tracker and Diary, Emotion, Life Robot- Fight Depression, Mood tracker- depression, Start
	Literature	1		iDepression Tracker
	Both	3		Depression Inventory, Depression Journal, Depression Test & Tracker
**CBT/BA**	Commercial Marketplace	4	12 (10.26)	MoodTools- Depression Aid, Overcome the Depression pro, Anti-Depression, Activity Diary
	Both	8		Depression, Depression CBT Self- Help Guide, Depression Cure- The free 12 week course, iCounselor: Depression, Mood Master Anti- Depression App, Mood Sentry, Positive Activity Jackpot, eCBT Mood
**State Induction**	Commercial marketplace	10	18 (15.38)	Beat Depression Hypnosis Audio, Depression Cure Hypnosis, Depression Mood Booster, Fight Depression, From Depression to Hope, MoodSpace, Vital Tones Depression, Yoga for Depression, Yoga Helps Relieve Depression, Life Robot- Fight Depression
	Literature	2		Depression Relief and Mood–HappyApp,Mood Elevator & Support
	Both	6		Beat Depression Hypnosis Syste, Depression Help Brainwave, Depression Inventory, Heal Depression Hypnosis, The Mindful Way Through Depression, Black Rainbow: How to Beat Depression
**Diagnostic/ screening**	Commercial Marketplace	15	30 (25.64)	CESD Depression Test, Depression Diagnosis Doctor, Depression Eval Questionnaire, Depression Screening Test, Depression Test, Depression Test, Depression Test, Depression Test, Depression Test, Depression Test, Depression Test and Treatment, Depression Tracker & Diary, Depression Test Pro, Depression Test, Emotion
	Literature	4		Am I Depressed, Do I have Depression, Happy App, Zung
	Both	11		Are You at Risk for Depression?, Depression Calculator, Depression Screening, Depression Test, Depression Test, Learn About Depression, Major Depression Checker, Sad Scale Lite, STAT Depression Screening PHQ 9, The Depression Predictor, Depression Test & Tracker
**Education**	Commercial Marketplace	22	32 (27.35)	Conquering Depression, Dealing with Depression, Dealing with Depression, Depression, Depression and How to Stop it, Depression & Psychology, Depression Definition, Depression Healing, Depression Information, Depression Management, Depression Symptoms, Depression Symptoms and Signs, Depression: An Overview, Depression: Natural Remedies, Fitness Against Depression, Help with Depression, How to get Over Depression, Physical Symptoms Depression, Reduce Depression, The Key to Happiness, Black Rainbow: How to Beat Depression, Emotion
	Literature	3		Beat Depression, Depression Treatment, Overcoming Depression
	Both	7		Depression 101, Depression Advice, NIH Depression Information, Ten Tips to Ease Depression, The Depression Predictor, Are You at Risk for Depression?, Depression Preview
**Social Support**	Commercial Marketplace	2	3 (2.56)	Dealing with Depression, You Are Important
	Both	1		Depression Test
**Unclear/ miscellaneous**	Commercial Marketplace	22	28 (23.93)	A Guiding Light, Acupuncture Against Depression, Afternoon in Depression, Best Depression Quotes, Depression, dePRESSION, Depression Quotes Wallpaper, Depression Quotes Wallpaper, Depression- Acupuncture, Depressive and Sad Wallpaper, Endless Depression, Get Rid of Depression with Chinese Massage Points, Guide to Depression Self- Help, How to Beat Depression, Sad Quotes Wallpaper, Sadness and Depression Quotes, Secret of Happiness, Self-Help for Depression, MoodSpace, Life Robot- Fight Depression, Depression Management, You are Important
	Literature	3		Depressed, DepressPill Game for Happy, Joker
	Both	3		Anti-Depression Grocery List, Depression Fighter- A Practical Christian Guide, Surviving Depression

^a^ The total sum of number of apps for each type of self-help app is not equal to the total number of apps identified through our searches (n = 117), since some apps have been categorized into multiple categories.

^b^ Percentage of the total 117 apps

### CBT/BA apps characteristics

Five of the 12 CBT/BA apps (41.67%) were available on iOS only and 5 (41.67%) on Android only. The cost of these CBT/BA apps ranged from $0.00 to $8.99. The Depression CBT Self-Help Guide and The Mood Tools–Depression Aid were those Android apps with the highest number of downloads (i.e., between 100,000 and 500,000 downloads, and between 50,000 and 100,000, respectively) and received high user satisfaction ratings (average satisfaction rates were 4.2 and 4.3, respectively). The iPhone app that received the highest user satisfaction rating was The Depression Cure: The Free 12 Week Course app (average satisfaction rating = 4.5). However, this app was not the one that received the highest number of reviews. The iPhone apps that received the highest number of reviews were the Anti-depression and MoodTools–Depression Aid apps, both of them also available for download in the Google Play store. For further information about the characteristics of the CBT/BA apps see Table 4.

**Table 4:** Currently-available CBT or BA apps for depression.

Name	Author	Commercial Market		Scientifc Literature	Cost	Popularity			Adherence with the core ingredients		Adherencewith the heuristics
		iTunes	Google Play			ASR^a^	# of reviews	# of downloads	CBT	BA	
Depression Cure: The free 12 week course	Archie’s Empire	✓	✗	✓	$8.99	4.5	29	n/a^b^	10%	18.75%	92%
iCounselor: Depression	iCounselor	✓	✗	✓	$1.19	2.5	9	n/a^b^	5%	6.25%	60%
Mood Master Anti depression App	Mood Master	✓	✗	✓	$4.59	n/a^b^	n/a^b^	n/a^b^	15%	18.75%	82%
eCBT Mood	MindApps LLC	✓	✗	✓	$1.19	2.5	12	n/a^b^	55%	25%	70%
Activity Diary	Happtic Pty. Ltd	✓	✗	✗	$3.49	n/a^b^	n/a^b^	n/a^b^	0%	18.75%	98%
Anti-depression	Dion LLC	✓	✓	✗	$0	3.7	250	10,000–50,000	25%	25%	88%
MoodTools—Depression Aid	MoodTools	✓	✓	✗	$0	4.3	1,466	50,000–100,000	10%	12.5%	98%
Mood Sentry	Mood Apps LLC	✗	✓	✓	$1.97	5.0	3	50–100	25%	6.25%	42%
Depression	AppCounselor	✗	✓	✓	$0.99	4.0	5	500–1,000	15%	6.25%	64%
Depression CBT Self-Help Guide	Excel at Life	✗	✓	✓	$0	4.2	1,154	100,000–500,000	75%	25%	62%
Overcome the Depression Pro	Zanapps	✗	✓	✗	$0	4.2	5	100–500	20%	18.75%	84%
Positive Activity Jackpot	T2	✗	✓	✓	$0	3.4	74	10,000–50,000	0%	12.5%	84%

^a^ ASR- Average Satisfaction Rating

^b^Some apps do not have enough user reviews to have an average rating, stated by n/a.

Regarding the validy and accuracy of the CBT/BA apps, the median level of adherence with the CBT principles was 15% (range = 0–75%) and the median level of adherence with the BA principles was 18.75% (range = 6.25–25%). The best apps from a theoretical perspective were Depression CBT Self-Help Guide and eCBT Mood meeting 75% and 55% of the qualifying criteria for CBT, respectively. The rest of the apps presented less than 50% of adherence for both the CBT and BA principles (see Table 5). The core ingredients of CBT most commonly included in these CBT/BA apps were: education about depression and depression ratings. The core ingredients included least often were: monitoring physical sensations, monitoring behaviors, and conceptualization. The core ingredients of BA most commonly included were: education about depression and depression ratings and the rest of the core ingredients were never completely integrated into the apps. Regarding the effectiveness of the apps, there were no studies reported in the scientific literature that determined the benefits of any of these CBT/BA apps.

**Table 5:** Evaluation of the usefulness dimension.

Usefulness Dimensions	Name												
	Depression Cure- the Free 12 Week Course	iCounselor: Depression	MoodMaster- Anti Depression App	eCBT Mood	Activity Diary	Anti-Depression	MoodTools-Depression Aid	Mood Sentry	Depression	Depression CBT Self-Help Guide	Overcome the Depression Pro	Positive Activity Jackpot	*Total*
Proven Efficacy	✗	✗	✗	✗	✗	✗	✗	✗	✗	✗	✗	✗	
**Core Ingredients of CBT**													
Education about depression	1	0	2	2	0	2	0	1	0	2	1	0	11
Explanation of the model	0	0	0	2	0	0	0	0	0	2	0	0	4
Depression rating	0	1	1	2	0	1	0	0	0	2	2	0	9
Monitoring cognitions	0	0	0	2	0	0	0	1	1	2	1	0	7
Monitoring emotions	0	0	0	1	0	0	0	1	0	2	0	0	4
Monitoring physical sensations	0	0	0	0	0	1	0	0	0	0	0	0	1
Monitoring behaviors	0	0	0	0	0	0	0	0	0	1	0	0	1
Conceptualization	0	0	0	1	0	0	0	0	0	1	0	0	2
Cognitive techniques	0	0	0	1	0	0	1	1	1	2	0	0	6
Behavioral techniques	1	0	0	0	0	1	1	1	1	1	0	0	6
*Total score out of 20 (%)*	2 (10)	1 (5)	3 (15)	11 (55)	0 (0)	5 (25)	2 (10)	5 (25)	3 (15)	15 (75)	4 (20)	0 (0)	
**Core Ingredients of BA**													
Education about depression	1	0	2	2	0	1	2	1	0	2	1	0	12
Explanation of the model	0	0	0	0	0	0	0	0	0	0	0	0	0
Depression rating	0	1	1	2	0	1	0	0	0	2	2	0	9
Activity monitoring	0	0	0	0	1	1	0	0	0	0	0	0	2
Activity monitoring: Pleasure rating	0	0	0	0	1	1	0	0	0	0	0	0	2
Activity monitoring: Masteryrating	0	0	0	0	1	0	0	0	0	0	0	0	1
Activity scheduling of pleasantbehaviors	1	0	0	0	0	0	0	0	1	0	0	1	3
Activity scheduling of avoidance behaviors	1	0	0	0	0	0	0	0	0	0	0	1	2
*Total score out of 16 (%)*	3 (18.75)	1 (6.25)	3 (18.75)	4 (25)	3 (18.75)	4 (25)	2 (12.5)	1 (6.25)	1 (6.25)	4 (25)	3 (18.75)	2 (12.5)	

The usability heuristic evaluation found that the median level of adherence with the heuristics was 83% (range = 42–98%). The apps associated with highest usability ratings were Mood Tools–Depression Aids, Activity Diary, and Depression on Cure–The Free 12 Week Course scoring 98%, 98%, and 92% respectively. The most frequent heuristic violations of these CBT/BA apps were: visibility of the system status, and consistency and standards. See Table 6.

**Table 6:** Evaluation of the usability dimension.

Name	Visibilityof system status	Match between system and the real world	User controland freedom	Consistency and standards	Error prevention	Recognition rather than recall	Flexibilityand efficiency of use	Aestheticand minimalist design	Help users recognize, diagnose, and recover from errors	Help and document-ation	*Total score out of 50(%)*
Depression Cure—The Free 12 Week Course	4	5	5	4	5	4	5	4	5	5	46 (92)
iCounselor: Depression	1	5	3	2	5	2	2	3	5	2	30 (60)
MoodMaster Anti-Depression App	3	5	4	3	5	4	4	4	5	4	41 (82)
eCBT Mood	2	5	4	2	5	2	3	5	5	2	35 (70)
Activity Diary	4	5	5	5	5	5	5	5	5	5	49 (98)
Anti- depression	4	5	4	5	5	5	3	4	5	4	44 (88)
Mood Tools–Depression Aids	5	5	4	5	5	5	5	5	5	5	49 (98)
Mood Sentry	2	4	3	2	1	2	2	3	1	1	21 (42)
Depression	2	5	3	2	5	2	2	3	5	3	32 (64)
Depression CBT Self-Help Guide	2	4	4	2	4	2	3	2	4	4	31 (62)
Overcome the Depression Pro	5	4	5	3	5	5	4	2	4	5	42 (84)
Positive Activity Jackpot	5	5	4	4	5	5	3	2	5	4	42 (84)
*Total*	39	57	48	39	55	43	41	42	54	44	

Only the eCBT app and the Depression CBT Self-Help Guide app offer a privacy policy. The eCBTapp has a brief privacy policy that states that the information collected in the app is only accessed by the application on the device and they do not collect any information about the user or the use of the app. The Depression CBT Self-Help Guide app’s privacy policy applies to this app in particular, but also other products of its developer (other apps and its homepage). This policy is available on the developer’s homepage but is also available to users after they have downloaded the app. Its privacy policy indicates what information is collected and for what purpose, whether this information is shared with others but it does not address users control. Five out of the 12 apps (41.66%) provide important safety information during crisis.See Table 7 for details about what information is provided and how.

**Table 7:** Evaluation of integration and infrastructure dimension.

Name	Do they have privacy policy?	Scope of privacy policy	Transparency of privacy policy				Do they deal with safety?	How?
			Type of information collected	Rationale for collection	Sharing of information	User control		
Depression Cure- the Free 12 Week Course	✗						✗	
iCounselor: Depression	✗						✗	
MoodMaster- Anti Depression App	✗						✗	
eCBT Mood	✓	Single app	✓	n/a^a^	n/a^a^	n/a^a^	✓	• If the user scores high on depression, they are encouraged to contact health care provider or crisis center (number is provided).
Activity Diary	✗						✗	
Anti-Depression	✗						✗	
MoodTools-Depression Aid	✗						✓	• Includes a safety plan feature, where user can input infomation about crisis warning signs, coping strategies, reasons to live, and add contacts to call. • There is also a “?” icon which gives the user the option to work with a therapist, allow user to visualize a safety plan video, and give them a direct link to call a help line. • There is a guide which goes through different stages from coping, to recovery, suicide prevention. • Static crisis tab with 4 different options; call 911, call helpline, and a map feature to either find urgent care or the nearest emergency department.
Mood Sentry	✗						✗	
Depression	✗						✓	• Once user provides a high rating of depression, a safety screen with information appears. • Static tab for the same safety screen appears within the learning module.
Depression CBT Self-Help Guide	✓	This app plus other apps created by the developer, and the homepage	✓	✓	✓	✓	✗	
Overcome the Depression Pro	✗						✓	• The user is encouraged to seek professional help if they score high on depression.
Positive Activity Jackpot	✗						✓	• In the license that user first sees upon enterting app, they make a brief statement about if user is in an emergency or life threatening situation to seek medical assistance or dial emergency number. • In the settings there is a statement that says “if at any point you feel suicidal please call crisis care hotline (number is provided).

^a^ All the information collected is stored on the device and can only be accessed by the user. Developer does not collect/store any information about the user or the use of the app.

No relationship was found between the level of adherence of the app to the theoretical CBT or BA model and the level of adherence with the heuristics usability (r_s_ = -0.45, p = 0.13 and r_s_ = 0.30, p = 0.33, respectively). Also, no relationship was found between level of adherence of the app to the theoretical models and the indicators of popularity (range = r_s_ = -0.02, p = 0.96 and r_s_ = 0.57, p = 0.18), or between level of adherence of the app with the heuristic usability with the indicators of popularity (range = r_s_ = 0.15, p = 0.68 and r_s_ = 0.59, p = 0.07).

## Discussion

While there are a large number of phone apps designed to assist those with depression available through the commercial market, few of these utilize a CBT or BA approach despite these being the gold standard of first line psychological treatments [72]. The few apps that provide CBT or BA seem to be popular based on the number of downloads, with 4 out of 7 of the Android available apps achieving more than ten thousand downloads.

Chan et al. [16] have recently proposed a framework that can be used for patients and health care providers to evaluate existing mental health mobile apps and help them make informed choices about their use. Chan et al. [16] suggest evaluating apps on three broad dimensions: usefulness, usability, and integration/infrastructure. After evaluating the usefulness dimension of the CBT/BA apps taking into account the main usefulness criteria of ‘effectiveness’, we can see that there is no available information on effectiveness. The few available apps that offer CBT or BA have either not been tested or the results derived from these tests have not been reported in the scientific literature. This means that we do not have any direct evidence demonstrating the efficacy of these CBT/BA apps and consequently we do not have direct scientific proof to support their use. All the apps identified through searching the scientific literature were simply cited in reviews [14]; they were not evaluated in primary research studies. Although no data on the efficacy of these CBT/BA apps have been published, we need to acknowledge that evidence may exist outside scientific journals. Knowledge can be disseminated through grey literature. The lack of direct scientific evidence for these CBT/BA apps, however, becomes especially alarming after evaluating the validity and accuracy of the content of these apps from an expert’s point of view. Of those apps which do use CBT or BA, some apps may provide benefits by partially applying CBT or BA principles, but the majority do not come close to including the core ingredients of a CBT or BA program. The lack of fidelity to proven CBT or BA principles could hamper the efficacy of these programs.

When evaluating the usability dimension, we have seen that the usability of the available CBT/BA apps is highly variable and likely serves as a barrier to adoption and regular usage for those apps that violate a large number of heuristics. For instance, the Depression CBT Self-Help Guide app has the highest fidely to CBT models, but the low usability score could complicate its use. There is a danger that users of these available CBT/BA apps may interpret ineffectiveness as a treatment failure, when in fact, ineffectiveness may be the result of usability problems or the inappropriate application of the CBT or BA model.

On the one hand, there doesn’t appear to be a correlation between CBT/BA model adherence and usability, which means that a good application of the clinical theoretical CBT or BA knowledge when designing the app does not imply a good use of principles of usability, and/or vice versa. On the other hand, the degree to which the apps contain these core ingredients of the CBT and BA models does not appear to be correlated with the extent to which users like the app, the number of downloads, or the number of reviews for the app. Equally, the level of usability of the CBT/BA apps does not appear to be correlated with the extent to which users like the app, the number of downloads or the number of reviews for the app. This finding is not surprising; previous reviews have found no relationship between the quality of the apps and consumers reviews or ratings [73,74]. Therefore, users should be careful when using the information available on the app download page to judge the app, since this information can be misleading.

When evaluating the integration and infrastructure dimension, we have seen that safety information is not always available in apps, and very rarely are users provided with a privacy policy. This lack of availability of privacy information seems to be an issue for mental health apps in general [21]. Research has shown that privacy is a concern for many health care professionals and patients [75] and this concern is a reason for them to decline the use information technology [75,76,77] as part of their care.

We have identified through our systematic review four apps in English that offer CBT or BA treatment for depression and have been studied by researchers and published in scientific papers, the Behavioural Activation Scheduling [50], the Get Happy Program [40], CBT Mobilwork [65] and Mobilyze [45]. However, these four apps have not been included in our full analysis because they are not currently available for download by the public, at least from within Canada. The lack of empirically tested apps identified during this review is consistent with observations in other health fields [36] and raises concerns about relying on these tools to support treatment for depression. We therefore launch a call for scientists and/or app developers interested in the opportunities that mobile communication technology offers in terms of improving access to mental health care to test the existing best apps and determine from the outset how to best implement and sustain the apps over time given that technology is evolving rapidly. It is also important when designing new CBT/BA apps to try to integrate the core ingredients of these theoretical models, and to address the heuristics in order to optimize clinical benefits and make the app more usable. Finally, it is important that scientists and developers are more transparent about legal and regulatory aspects of the apps related to privacy issues (e.g., [78]). Failure to effectively plan for sustainable dissemination of apps as well as the lack of consideration of legal aspects may present significant barriers for using apps.

This review is not without limitations. First, this review was limited to English downloadable apps in Canada and only looked at the two most popular platforms when exploring the commercial market. Different apps may be available on less prevalent platforms or in other languages and/or countries, and in fact we excluded apps developed and tested in the academic setting for these reasons[9,40]. Second, the evaluation of the CBT and BA apps was based on the opinion of one expert. Although expert opinion plays an important role when no research evidence exists, the use of an expert panel instead of only one expert could have increased the credibility of the conclusions. Finally, although it was not the primary goal of this review, the lack of common constructs, outcome measures, definitions and/or standards for tracking, state induction, diagnostic/screening, and education apps make cross-case comparison of these different types of self-help apps impossible.

In summary, given the prevalence of depression [1] and the known effectiveness of CBT and BA in addressing this mental health condition [6,7], a mobile app based on clinical best practice, that meets the most basic usability standards, that is evaluated scientifically, has a privacy policy, and deals with safety matters has the potential to remove barriers to care and alleviate suffering for a large number of people with depression at a modest cost. Therefore, efforts towards achieving this are necessary.

## Supporting Information

S1 PRISMA Checklist PRISMA 2009 Checklist. (DOCX)

S1 Appendix Search strategy used for Pubmed. (DOCX)
