# Supplementary material for: A Systematic Review of Cognitive Behavioral Therapy and Behavioral Activation Apps for Depression
Source: PLoS One. 2016 May 2;11(5):e0154248. doi: 10.1371/journal.pone.0154248 (PMC4852920; doi:10.1371/journal.pone.0154248)
Supplement: S1 Appendix — (DOCX) [file pone.0154248.s002.docx]

**S1 Appendix.** **Search strategy used for Pubmed.**

(("Depression"[Mesh] OR "Depressive Disorder"[Mesh]) OR (depress*[tiab])) AND (((((((((((((((((((("Mobile handset"[tiab] OR "mobile handsets"[tiab])) OR (short messag*[tiab])) OR (cell phon*[tiab])) OR (cellular phon*[tiab])) OR (mobile phon*[tiab])) OR (mobile handset*[tiab])) OR (personal digital assistant*[tiab])) OR (smartphone*[tiab])) OR (mobile technolog*[tiab])) OR (android*[tiab])) OR (apps[tiab])) OR (iphone*[tiab] OR ipad*[tiab] OR ipod*[tiab]))) OR (mobile telephon*[tiab])) OR (mobile devic*[tiab])) OR (mobile technolog*[tiab])) OR (m-health[tiab])) OR ("mobile health"[tiab])) OR text messag*[tiab] OR ("Cellular Phone"[Mesh]))
